# High Environmental Irradiance Induces Anatomical and Ultrastructural Damage in *Citrus* Leaves

**DOI:** 10.1111/ppl.70901

**Published:** 2026-04-21

**Authors:** Lucas Giovani Pastore Bernardi, João Paulo Rodrigues Marques, Gabriel Antonio Bortoloti, Rodrigo Marcelli Boaretto, Dirceu Mattos

**Affiliations:** ^1^ Sylvio Moreira Citrus Research Center—Agronomic Institute (IAC) Cordeiropolis SP Brazil; ^2^ Laboratory of Applied Botany, Department of Basic Sciences, School of Animal Science and Food Engineering University of São Paulo (USP) Pirassununga SP Brazil

**Keywords:** abiotic stress, calcium carbonate, cuticle, irradiation, kaolin, particle films

## Abstract

Climate change is intensifying episodes of high irradiance and thermal stress, posing a major threat to the sustainability of citrus production. Although reflective particle films have emerged as mitigation tools, their protective effects at the anatomical and ultrastructural levels under field conditions remain insufficiently understood. Here, sweet orange plants ( *Citrus sinensis* (L.) Osbeck cv. Valencia) were exposed to full sunlight, shaded or treated with kaolin or calcium carbonate particle films, and leaves were examined using light and electron microscopy, alongside foliar nutrient analysis. Exposure to full sunlight caused disruption of thylakoid membranes and mitochondrial ultrastructural alterations consistent with stress‐associated metabolic imbalance, increased plastoglobule accumulation, and alterations in oil cavities, indicating sustained photo‐oxidative pressure. These effects were accompanied by marked anatomical changes, including cell hyperplasia and reduced intercellular air spaces, together with elevated foliar K and Mg concentrations that may reflect stress‐driven changes in leaf structure and ion homeostasis. In contrast, both particle film treatments preserved cellular and organelle integrity comparable to that of shaded plants, demonstrating that the structural injury observed under full sunlight is directly attributable to excess irradiance. This study provides mechanistic evidence of the limits of citrus leaf structural tolerance to high irradiance and establishes a detailed anatomical and ultrastructural baseline to support the development of more effective mitigation strategies under adverse field climate conditions.

## 1 Introduction

Citrus orchard productivity has been compromised by extreme climate events in recent years, with losses exceeding 20 million boxes (40.8 kg each) of oranges in the Brazilian citrus belt in the 2024/2025 harvest compared with 2023/2024 (Fundecitrus 2025). These reductions have been associated with elevated solar irradiance and high air temperatures during critical stages of flowering and fruit set, leading to substantial fruit losses (Bernardi et al. 2023; Cong et al. 2025). High irradiance, coupled with low soil water availability, increases leaf heat load, raising leaf temperatures above ambient levels during peak heat hours (Neves et al. 2019; Jain et al. 2025). As net carbon assimilation in citrus trees declines above the optimum temperature (around 30°C; Syvertsen and Smith‐Jr 1984), mitigating excess leaf temperature and radiative load poses a critical challenge for citrus production systems.

Beyond stomatal regulation, plant responses to excessive heat and irradiance involve structural adaptations such as changes in cuticle thickness and modifications in the anatomy of the palisade and spongy parenchyma (Trivedi et al. 2019; Samat et al. 2025). High light intensity can impair thylakoid membranes and disrupt organelle organization (including mitochondria), while increasing plastoglobule abundance, all key features associated with altered photosynthetic function (Zechmann 2019; Tang and Zhu 2022). Plastoglobules play pivotal roles in thylakoid membrane repair and in supporting the development and stabilization of photosynthetic structures (Rottet et al. 2015; Espinoza‐Corral et al. 2021). Concurrently, nutrient allocation, particularly of potassium (K) and magnesium (Mg), contributes to the adaptive response to high irradiance and heat stress (Ahammed et al. 2022).

To mitigate these environmental stressors, several agricultural strategies have been proposed, including the application of reflective particle films to leaf surfaces (Abd El‐Naby et al. 2020; Gullo et al. 2020). Composed primarily of either kaolin (Al_2_Si_2_O_5_(OH)_4_) or calcium carbonate (CaCO_3_), these films act as light‐reflective barriers when sprayed onto the canopy (Bernardi et al. 2023). However, a critical gap remains in the integrated anatomical and ultrastructural evidence required to identify which specific cellular and organellar injuries represent the primary limitations to citrus productivity under high environmental irradiance. Comprehensive mechanistic studies linking thylakoid and mitochondrial degradation with compensatory physiological responses are essential for establishing a structural baseline that defines the adaptation limits of *Citrus*.

This study aimed to establish a comprehensive anatomical and ultrastructural baseline of the cellular damage induced by excess solar irradiance in sweet orange plants. We exposed plants to full sunlight (control), particle film treatments (kaolin or CaCO_3_), and physical shading, and used microscopy and nutrient analyses to characterize the structural hierarchy of the damage. We hypothesized that excess irradiance induces irreversible structural damage, and that preventing this energy overload with particle films or shading helps maintain stable cellular architecture.

## 2 Materials and Methods

### 2.1 Plant Material and Growing Conditions

The experiment was conducted at the Centro de Citricultura Sylvio Moreira (22°27′40″ S, 47°24′4″ W; 639 m a.s.l.) using young sweet orange plants ( *Citrus sinensis* (L.) Osbeck cv. Valencia) grafted onto ‘Swingle”’ citrumelo ( *Citrus paradisi* Macf. × *Poncirus trifoliata* (L.) Raf.). Plants were grown in 12 L pots filled with an organic substrate and supplemented with mineral fertilizers according to crop requirements. They were maintained under drip irrigation to avoid drought stress throughout the study. In September 2020, four months before the experiment began, trees were pruned to promote uniform shoot growth. Treatments were applied in January 2021, marking the start of the 60‐day experimental period, which ended in March.

The experiment followed a completely randomized design with six replicates per treatment, each replicate consisting of one plant. The treatments were: (1) full sunlight sprayed with water (control); (2) full sunlight sprayed with kaolin (wettable powder, 1 μm, 30 g L^−1^); (3) full sunlight sprayed with calcium carbonate (wettable powder, < 1 μm, 30 g L^−1^); (4) plants covered with aluminum shade cloth (Aluminet, 50% shade); and (5) plants covered with low‐density polyethylene (LDPE) transparent plastic with an anti‐UV property (> 80% light transmissivity; 150 μm thickness). Sprays were applied using a manual pressure sprayer for 15 s per plant at a flow rate of 220 mL min^−1^. Kaolin and calcium carbonate were applied as aqueous suspensions (30 g L^−1^) prepared from commercial wettable powders, homogenized immediately before spraying. Sprays were directed uniformly to the canopy until uniform coverage of the leaf surfaces. Control plants and those under the aluminum shade cloth or anti‐UV plastic were sprayed with water only.

Plants were placed on benches 50 cm above the ground, either under full sunlight or inside the covered structures. Custom 5 × 6 m frames were built for the covered treatments to prevent shadow overlap and allow free air circulation. Air temperature (*T*_air_; 29°C ± 0.05°C) and relative humidity (RH; 59.4% ± 0.09%) were continuously monitored at mid‐canopy height (1.0 m above ground) using automatic sensors (S‐LIA‐M003, HOBO Data Logger Solution; Cordeiro‐Jr et al. 2021). Under full sunlight, midday PAR (12:00–13:00 h) reached 1557–1969 μmol m^−2^ s^−1^ during the 60‐day experimental period. *T*_air_ and RH did not differ between open and covered treatments during the experimental period.

### 2.2 Evaluation of Particle Film Reflectivity

Leaf surface reflectivity (L*) was measured using a spectrophotometer (CR‐300; Minolta) to assess the efficacy of the particle film coatings. One leaf per replicate was used (six leaves per treatment). L* values (0 = black, 100 = white) correspond to the colorimetric lightness/whiteness index and were used as a proxy for particle film coverage and surface optical change. These values were recorded on the adaxial leaf surface of clean leaves before application and 24 h after kaolin or calcium carbonate spraying.

### 2.3 Mineral Element Distribution and Leaf Anatomy

At the end of the experimental period, leaf sampling was performed between 09:00 and 11:00 h to analyze mineral element distributions and leaf anatomy. Fully expanded leaves that were already present on the plants at the beginning of the treatments were selected. For each plant, eight leaves were collected and arranged into four pairs of opposite leaves, with each pair taken from one of the four canopy sectors.

#### 2.3.1 Light Microscopy

Leaf samples were fixed in Karnovsky's solution (Karnovsky 1965) and embedded in Historesin (Marques and Nuevo 2022). Dehydration was performed through an ascending ethanol series, followed by infiltration with Technovit resin. The blocks were sectioned at 2–7 μm using a Leica microtome, mounted on glass slides, and subsequently stained with toluidine blue (Sakai 1973) for standard histological analysis. Histochemical tests were also carried out to detect cuticular, phenolic, and lipophilic compounds (Marques and Soares 2022). Analyses were conducted using a ZEISS Axio microscope.

#### 2.3.2 Scanning Electron Microscopy

Leaf samples were processed following Beltrame et al. (2025). Samples were fixed in Karnovsky's solution (Karnovsky 1965), dehydrated in an ascending acetone series (10%–100%), and dried using CO_2_ critical point drying (Horridge and Tamm 1969). Samples were mounted on aluminum stubs using double‐sided carbon tape and coated with a 30‐nm carbon layer.

An EDS detector was used to generate chemical maps of the spatial distribution of coating particles on citrus leaves. Carbon‐coated leaf tissues were examined at 20 kV and a working distance of 10 mm using a JEOL IT300 scanning electron microscope equipped with an EDS x‐ray detector.

### 2.4 Foliar Nutrient Concentration

Eight leaves per plant were collected 60 days after treatment for macro‐ and micronutrient analysis. Leaves were washed with a 5% detergent solution followed by distilled water (Alva and Tucker 1997), dried in a forced‐air oven to constant mass, and ground in a Wiley mill. Element concentrations were determined by chemical analysis according to the method of Bataglia et al. (1983).

### 2.5 Statistical Analysis

Leaf reflectivity, cuticle thickness, and nutrient concentrations were subjected to analysis of variance (ANOVA). When significant differences were detected, means were compared using Tukey's test at the 5% significance level.

## 3 Results

### 3.1 Reflectivity and Distribution of Coating Particles

**FIGURE 1:**
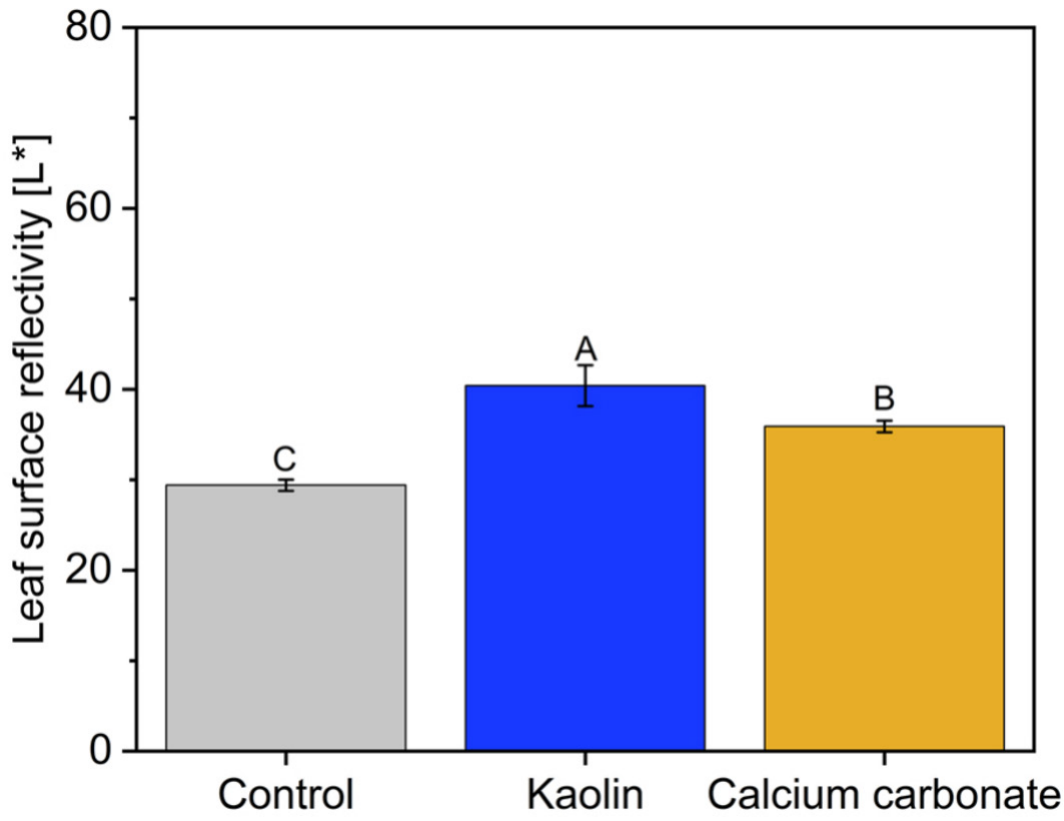
Particle film–induced changes in leaf surface reflectivity. Leaf surface reflectivity (L*, 0 = black, 100 = white) of sweet orange ( *Citrus sinensis* (L.) Osbeck cv. Valencia) leaves under different treatments: control (water‐sprayed), kaolin (wettable powder; 30 g L^−1^), and calcium carbonate (wettable powder, < 1 μm, 30 g L^−1^) application. Measurements were taken on the adaxial leaf surface (*n* = 6 per treatment). Bars represent means ± SE; different letters indicate significant differences (Tukey's test, *p* ≤ 0.05).

The reflective properties of the particle films were evident, with leaf reflectivity approximately 30% higher in plants sprayed with kaolin and 20% higher in those sprayed with calcium carbonate compared to water‐sprayed control leaves (Figure 1).

**FIGURE 2:**
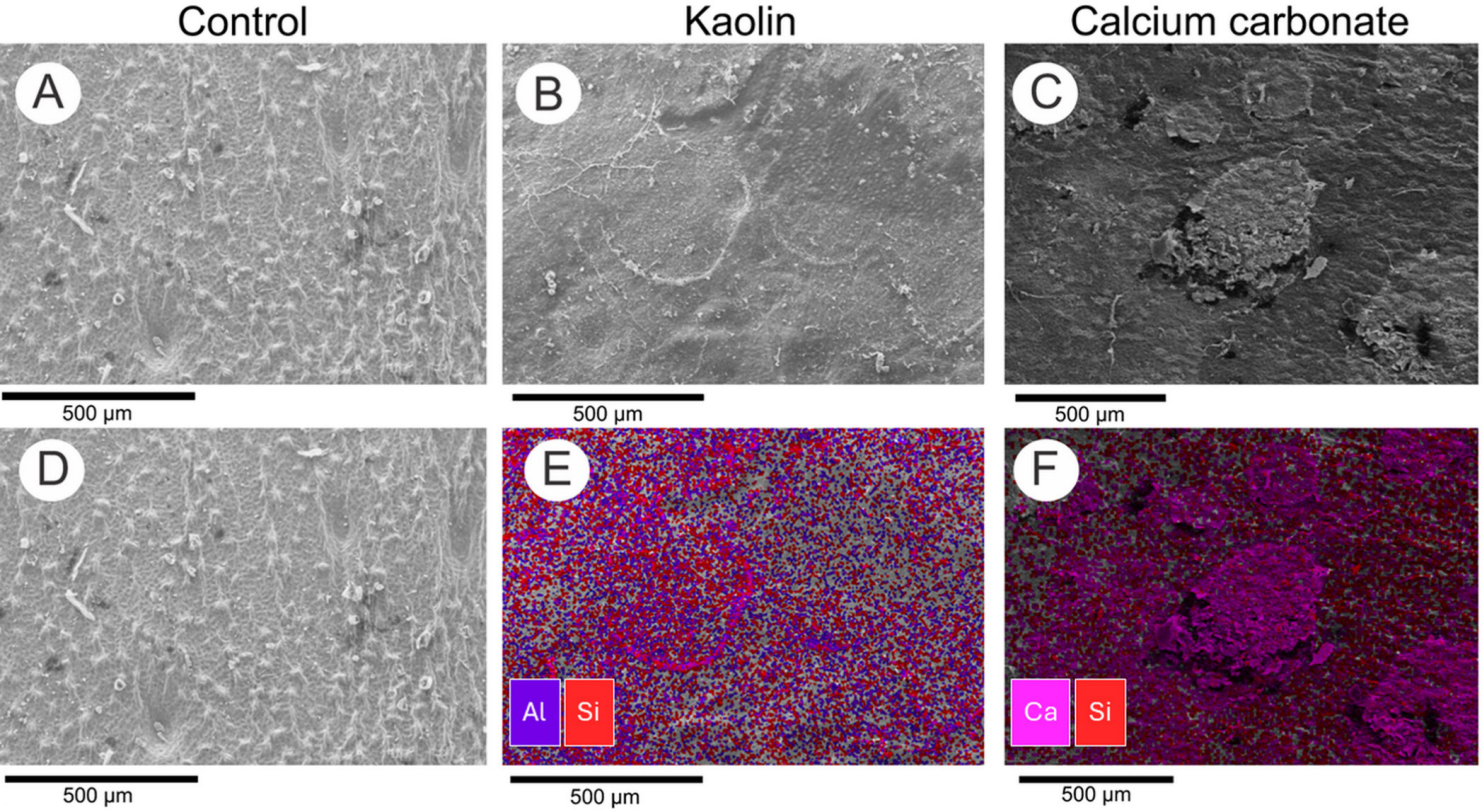
Surface deposition patterns of particle films on sweet orange leaves. Scanning electron microscopy (SEM) images (A–C) and energy‐dispersive x‐ray spectroscopy (EDX) distribution maps (D–F) of mineral elements on the adaxial surface of sweet orange ( *Citrus sinensis* (L.) Osbeck cv. Valencia) under different treatments. (A, D) control (water‐sprayed), (B, E) kaolin (wettable powder, 1 μm, 30 g L^−1^), and (C, F) Calcium carbonate (wettable powder, < 1 μm, 30 g L^−1^). Calcium (pink), aluminum (purple), and silicon (red) signals are shown. Images and maps were obtained using a JEOL IT 300 SEM with EDX detector at 20 kV and 10 μm working distance (*n* = 6 per treatment). Scale bar: 500 μm (applies to all panels).

Analysis of particle distribution provided insights into the protective potential of the coatings (Figure 2). EDX imaging of control leaves exhibited no deposition of coating particles (Figure 2A). In contrast, leaves sprayed with kaolin or calcium carbonate exhibited marked particle accumulation (Figure 2B,C). Kaolin formed finer, more evenly dispersed particles across the leaf surface, whereas calcium carbonate produced thicker, agglomerated deposits (Figure 2B,C). EDX–SEM analysis confirmed that kaolin‐treated surfaces contained high atomic percentages of silicon (Si, ~55%) and aluminum (Al, ~34%; Figure 2E), while calcium carbonate‐treated surfaces displayed high calcium content (Ca, ~80%; Figure 2F). As expected, no coating particles were detected on control leaves (Figure 2A,D).

### 3.2 Light Microscopy and Anatomical Structures

**FIGURE 3:**
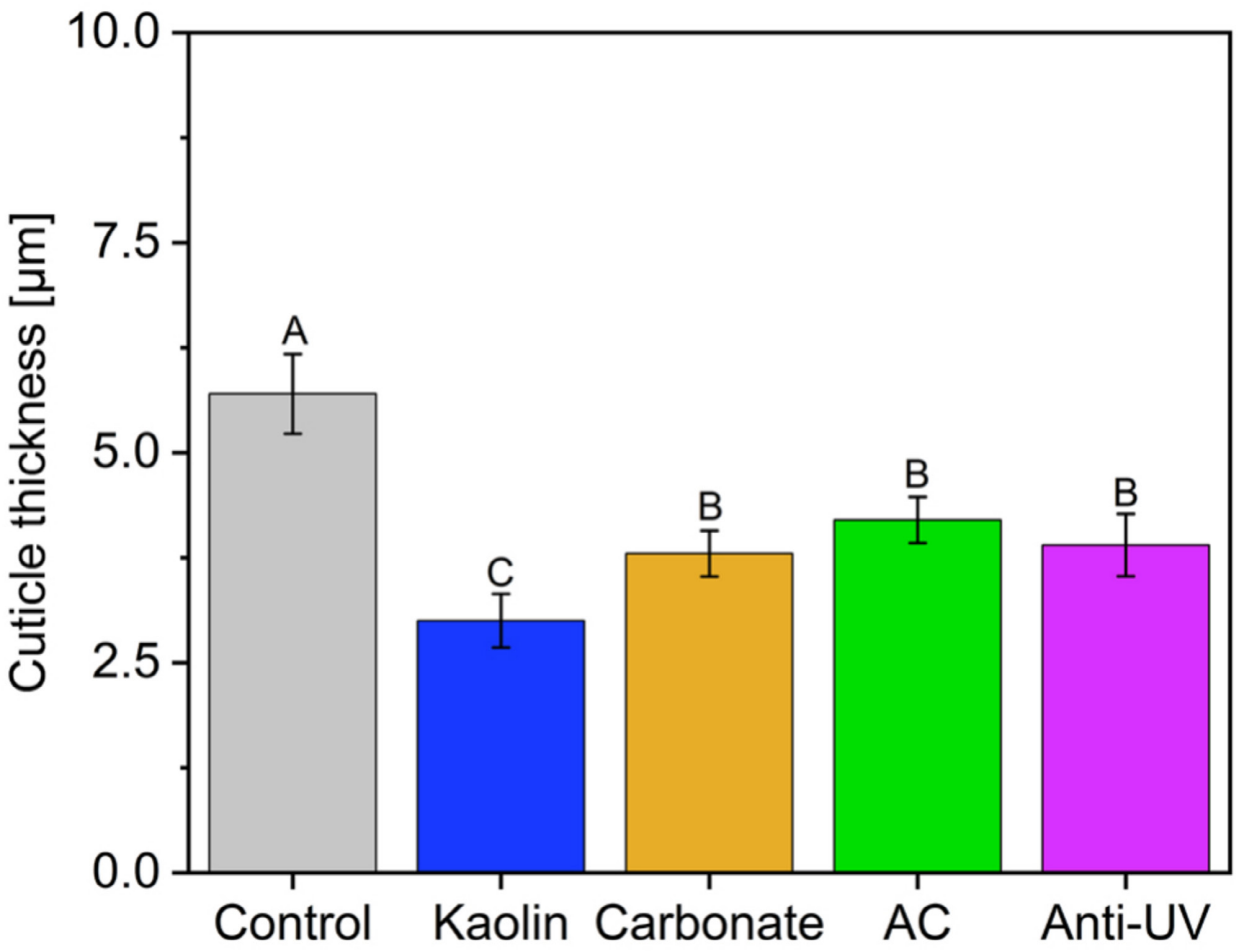
Cuticle thickening under full sunlight and protective treatments. Cuticle thickness (μm) of sweet orange [ *Citrus sinensis* (L.) Osbeck cv. Valencia] leaves under different treatments: Control (water‐sprayed), Kaolin (wettable powder, 30 g L^−1^), calcium carbonate (wettable powder, 30 g l^−1^), aluminum shade cloth (Aluminet, 50% shade), plants covered with low‐density polyethylene (LDPE) transparent plastic with an anti‐UV (anti‐UV; > 80% light transmissivity; 150 μm thickness). Measurements were taken on adaxial leaf surfaces (*n* = 6 per treatment) using light microscopy of histological sections fixed in Karnovsky's solution. Bars represent means ± SE; different letters indicate significant differences (Tukey's test, *p* ≤ 0.05).

**FIGURE 4:**
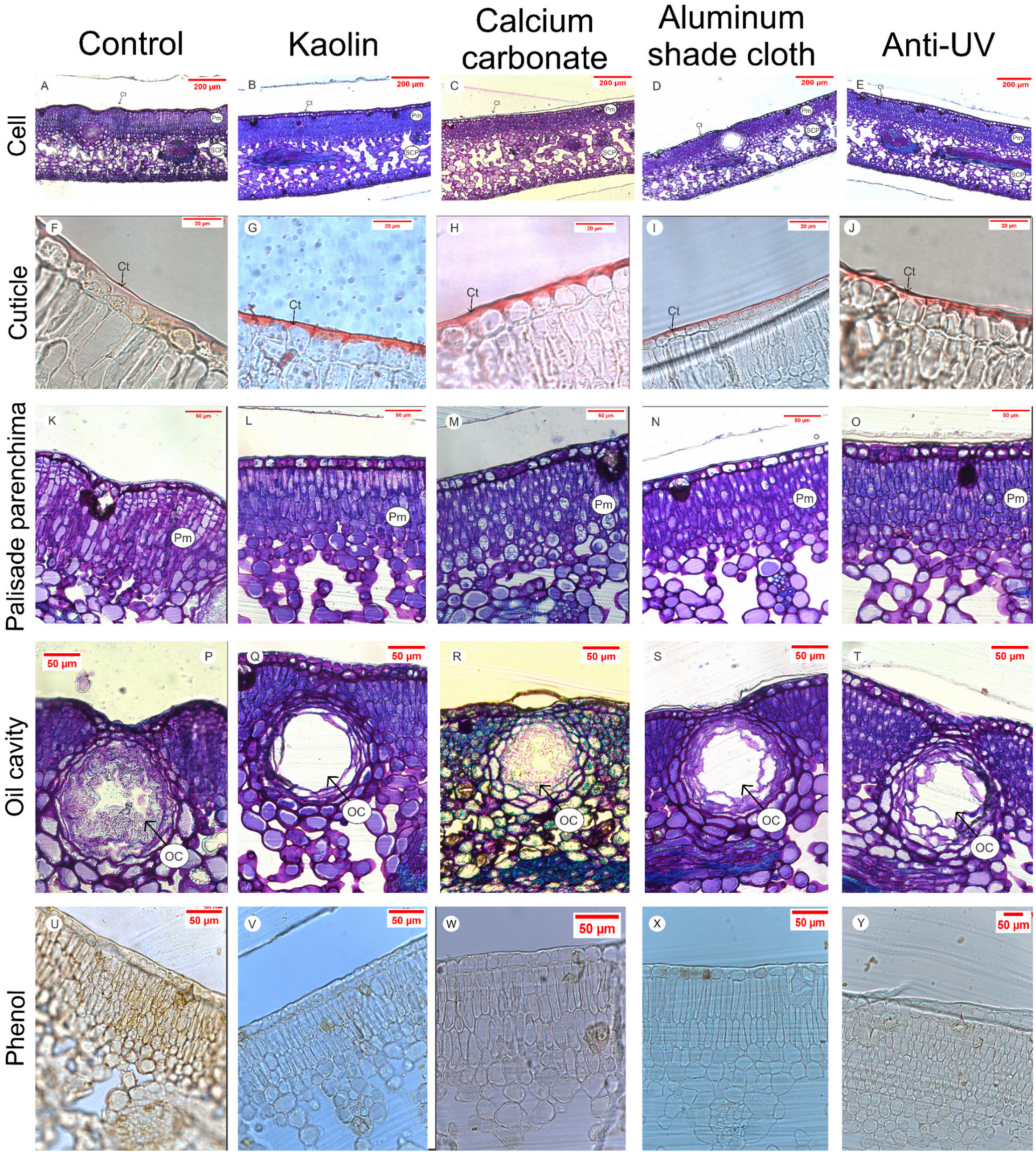
Anatomical responses of sweet orange leaves to full sunlight and protective treatments. Light microscopy images of transverse sections of sweet orange ( *Citrus sinensis* (L.) Osbeck cv. Valencia) under different treatments: Control (water‐sprayed), Kaolin (30 g L^−1^), Calcium carbonate (30 g L^−1^), aluminum shade cloth (Aluminet, 50% shade) and anti‐UV plastic (LDPE). (A–E) Leaf overview stained with toluidine blue; (F–J) Cuticle (Ct; Sudan IV); (K–O) palisade parenchyma (Pp) details; (P–T) oil cavity (OC) details; (U–Y) ferric chloride test for phenolic compounds (Phenolics). Samples (*n* = 6 per treatment) were fixed in Karnovsky's solution and sectioned at 2–7 μm using a Leica microtome and observed with a ZEISS Axion microscope. Scale bars: 200 μm (A–E), 20 μm (F–J), 50 μm (K–Y).

Anatomical differences were evident among treatments (Figures 3 and 4). Control leaves developed a cuticle that was 1.9‐fold thicker than that of kaolin‐sprayed plants and approximately 1.7‐fold thicker than that of calcium carbonate–sprayed plants (Figure 3). Plants grown under aluminum shade cloth or anti‐UV plastic exhibited cuticle thickness similar to that of calcium carbonate‐sprayed leaves (Figure 3).

Leaves from control plants exhibited irregular formation of the adaxial surface (Figure 4A). Regarding internal structures, plants treated with calcium carbonate, kaolin, aluminum shade cloth, or anti‐UV plastic did not show hyperplasia in palisade parenchyma cells (Figure 4L–O). Leaves from kaolin‐ and calcium carbonate–treated plants, as well as those grown under anti‐UV plastic, displayed thicker palisade cells and a greater number of palisade parenchyma layers compared to the control and aluminum shade cloth treatments. This arrangement led to larger, more visible intercellular air spaces in the spongy parenchyma.

**FIGURE 5:**
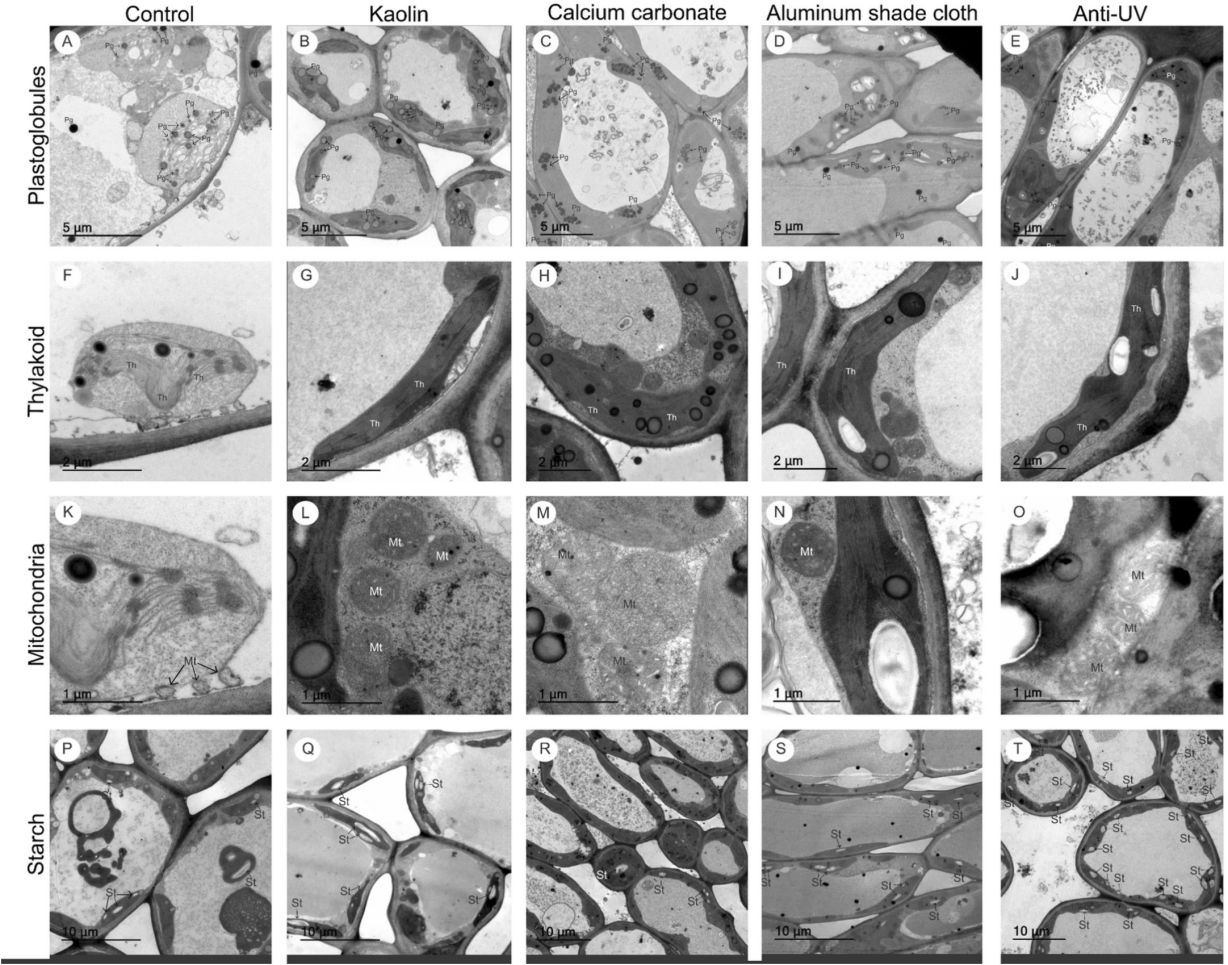
Ultrastructural responses of sweet orange leaves to full sunlight and protective treatments. Transmission electron microscopy (TEM) images of leaf ultrastructure in sweet orange ( *Citrus sinensis* (L.) Osbeck cv. Valencia) under different treatments: Control (water‐sprayed), Kaolin (30 g L^−1^), Calcium carbonate (30 g L^−1^), aluminum shade cloth (Aluminet, 50% shade), and anti‐UV plastic (LDPE). Panels show: (A–E) plastoglobules (Pg); (F–J) thylakoids (Th); (K–O) mitochondria (Mt); (P–T) starch grains (St). Samples (*n* = 6 per treatment) were fixed in Karnovsky's solution, dehydrated in acetone and examined by TEM. Scale bars: 5 μm (A–E), 2 μm (F–J), 1 μm (K–O), and 10 μm (P–T).

Oil cavities in control leaves exhibited irregular shapes, with wrinkled epithelial cell walls and reduced lumens compared with the other treatments (Figure 5P–T). Additionally, phenolic compounds, visible as brown‐stained droplets within the cells, accumulated exclusively in control plants exposed to full sunlight (Figure 5U–Y), whereas they were absent or minimal in the other treatments.

Plastoglobules were present in all treatments, but their abundance and degree of agglomeration were more pronounced in leaves from control plants and those treated with calcium carbonate compared with leaves treated with kaolin or plants grown under aluminum shade cloth or anti‐UV plastic (Figure 5A–E).

Marked alterations in chloroplast ultrastructure were evident in control plants under full sunlight, where thylakoid membranes appeared ruptured and disorganized (Figure 5F). In contrast, leaves of plants sprayed with kaolin or calcium carbonate, as well as those grown under aluminum shade cloth or anti‐UV plastic, maintained a well‐preserved and linear thylakoid organization (Figure 5G–J). Mitochondria in control leaves exhibited irregular, reduced morphologies, in contrast to the typical oval shape observed in all other treatments (Figure 5K–O).

Differences in carbohydrate storage were also evident. Kaolin‐treated leaves accumulated larger starch grains (Figure 5Q), whereas plants grown under aluminum shade cloth or anti‐UV plastic exhibited higher numbers of starch grains of intermediate size (Figure 5S–T). By contrast, control leaves, together with those sprayed with calcium carbonate, accumulated the lowest amounts of starch reserves (Figure 5P,R).

### 3.3 Leaf Mineral Nutrient Concentration

Leaf concentrations of K, Mg, boron (B), and iron (Fe) differed among treatments (Table 1). Control plants exposed to full sunlight exhibited 22% higher K concentrations compared with plants sprayed with kaolin or calcium carbonate. Similarly, Mg concentrations were 16% higher in leaves of plants grown under aluminum shade cloth than in those treated with kaolin or calcium carbonate. The highest B and Fe concentrations were observed in control plants and in plants grown under aluminum shade cloth. Leaf concentrations of N, P, Ca, S, Mn, Cu, and Zn did not differ among treatments.

**TABLE 1:** Leaf mineral nutrient concentrations in young sweet orange ( *Citrus sinensis* (L.) Osbeck cv. Valencia) trees exposed to full sunlight: Control (sprayed with water) or sprayed with kaolin or calcium carbonate, and in trees grown under reduced irradiance using aluminum shade cloth or anti‐UV plastic.

Treatment	N (g kg^−1^)	P (g kg^−1^)	K (g kg^−1^)	Ca (g kg^−1^)	Mg (g kg^−1^)	S (g kg^−1^)	B (mg kg^−1^)	Fe (mg kg^−1^)	Mn (mg kg^−1^)	Cu (mg kg^−1^)	Zn (mg kg^−1^)
Control	35.2	1.5	20.2a	28.1	3.1ab	2.3	93a	86a	79	80	22
Kaolin	36.2	1.4	15.7b	25.8	2.7b	2.0	79ab	62b	69	69	20
Calcium carbonate	37.1	1.4	16.1b	26.1	2.8b	2.4	83ab	69b	76	76	17
Aluminum shade cloth	35.2	1.5	18.1ab	28.1	3.3a	2.4	90a	90a	69	67	17
Anti‐UV	33.9	1.5	18.9ab	23.2	3.1ab	2.4	75b	77ab	74	74	18
*F* test	NS	NS	**	NS	**	NS	**	**	NS	NS	NS

*Note:* Same letters do not differ from each other by the Tukey's test at 5% statistical probability.

Abbreviations: Aluminum shade cloth = reduced irradiance under aluminum shade cloth (Aluminet, 50% shade); Anti‐UV = low‐density polyethylene type (LDPE) transparent plastic (anti‐UV additive, > 80% transmissivity), 150 μm thick; Calcium carbonate = full sunlight sprayed with calcium carbonate (wettable powder, < 1 μm (30 g L^−1^)); Control = full sunlight sprayed with water; Kaolin = full sunlight sprayed with kaolin (wettable powder, 1 μm (30 g L^−1^)); NS = not significant.

** *p* ≤ 0.05, *F* test.

## 4 Discussion

The integrity of cellular organelles was assessed through visual examination to detect irreversible damage and functional impairment—key mechanistic indicators supporting the physiological and biochemical responses previously reported for citrus under high irradiance (Bernardi et al. 2023). In the full‐sun environment, incident midday PAR, monitored at mid‐canopy height, reached 1557–1969 μmol m^−2^ s^−1^ during the 60‐day experimental period. Under these irradiance levels, the thylakoid degradation observed in full‐sun control plants is consistent with photoinhibitory stress driven by thylakoid overexcitation, whereas this disruption was absent in leaves sprayed with kaolin, demonstrating the reflective protection provided by this particle film.

This protective effect is further supported by the similarity in thylakoid organization between kaolin‐treated plants and those grown under aluminum‐cloth shade. A comparable pattern of thylakoid organization was also observed in calcium carbonate‐treated plants (Figure 5H) and in plants grown under anti‐UV plastic (Figure 5J).

The maintenance of thylakoid membrane structure is essential for sustaining photosynthetic function and resilience to high‐light stress. Kaolin application has been shown to improve the operating efficiency of Photosystem II (PSII) and enhance nonphotochemical quenching (NPQ) under high environmental irradiance (Bernardi et al. 2023). The preservation of thylakoid organization observed in kaolin‐treated plants aligns with this enhanced photoprotective capacity. In contrast, the disorganized thylakoid membranes of control plants indicate structural impairment of the photosynthetic apparatus, limiting both photochemical conversion and the dissipation of excess energy via NPQ. Thus, thylakoid degradation reflects the collapse of the photosynthetic machinery under excess‐irradiance stress (Sakamoto 2025).

Maintaining photosynthetic function under high irradiance depends not only on thylakoid integrity but also on cellular energy and redox balance. Chloroplast–mitochondrion interactions contribute to this balance, as mitochondrial respiration and redox exchange mechanisms can consume excess reducing equivalents when light absorption exceeds carbon assimilation. Thus, the altered mitochondrial morphology observed in full‐sun control leaves—notably reduced size and irregular morphology—likely reflects stress‐induced metabolic imbalance accompanying chloroplast photoinhibition. In contrast, the typical mitochondrial morphology in protected treatments (Figure 5L,M) is consistent with improved cellular homeostasis when irradiance load on chloroplasts is mitigated (Selinski et al. 2024; Monteiro‐Batista et al. 2025).

Structural injury is tightly linked to the overproduction of reactive oxygen species (ROS) in plants (Bernardi et al. 2023). Excessive ROS levels can trigger plastoglobule proliferation as a defensive response. Plastoglobules were detected in all treatments but were most abundant and highly agglomerated in full‐sun control plants and calcium carbonate‐treated plants. Although calcium carbonate preserved thylakoid organization, its thicker and more agglomerated deposition pattern likely reduced the uniformity of light scattering on the leaf surface, allowing continued ROS formation and lipid remodeling, consistent with plastoglobule proliferation as a marker of thylakoid membrane turnover (Rottet et al. 2015; van Wijk and Kessler 2017).

The overproduction of ROS and resulting oxidative stress compromise membrane stability and impair cellular function. Although calcium carbonate‐treated plants maintained the overall organization of the cuticle and parenchyma, the oil cavities in these leaves retained characteristics like those of the full‐sun control plants. Bernardi et al. (2023) reported higher malondialdehyde (MDA) levels, an indicator of lipid peroxidation caused by membrane damage, in calcium carbonate‐treated plants compared with plants grown under anti‐UV plastic. These findings reinforce the idea that the increase in oil cavities, or lipid compartments, is linked to oxidative stress and membrane degradation.

Plants rely on both enzymatic and nonenzymatic systems to mitigate excess ROS (Soares et al. 2019). Phenolic compounds, visible as brown droplets in leaf sections of control plants (Figure 5), play a key antioxidant role by scavenging ROS and restoring redox balance (Mishra et al. 2023). Additionally, phenolic compounds act as natural filters of PAR and UV radiation, mitigating damage resulting from excessive environmental irradiance (Gambetta et al. 2021).

Leaf tissue modification is a typical response to heat and high irradiance (Zhang et al. 2022). Control plants produced a markedly thickened cuticle (Figures 3 and 4F), often associated with enhanced wax deposition in high‐light environments (Liu, Wang, and Chang 2022; Tomasi et al. 2024). In contrast, the thinner cuticle observed in particle film‐coated leaves reflects their protective role in reducing direct irradiance exposure (Figures 3 and 4G–J).

Leaves of full‐sun control plants and those grown under aluminum shade cloth had fewer palisade parenchyma layers than leaves from the other treatments (Figure 4K–O). In plants grown under aluminum shade cloth, the reduced number of palisade layers likely reflects the lower irradiance environment created by the reflective shade cloth, which diminishes the developmental stimulus for palisade differentiation and elongation. While aluminum shade cloth reduces thermal and oxidative stress, the markedly reduced light availability limits palisade development, whereas kaolin, calcium carbonate, and anti‐UV plastic treatments reduce excess irradiance without entirely suppressing incoming light.

Control plants also exhibited palisade cell hyperplasia and reduced intercellular air spaces in the spongy parenchyma. In contrast, particle film‐treated leaves displayed more organized cellular structure and larger intercellular air spaces. Such an organization is crucial for enhancing photosynthetic capacity and RuBisCO activity by facilitating CO_2_ diffusion within the leaf (Evans et al. 2009; Terashima et al. 2011; Théroux‐Rancourt et al. 2023).

Differences in carbohydrate storage were also evident. Overall, treatments associated with stronger photoprotection exhibited greater starch reserves, whereas control and calcium carbonate leaves accumulated less starch. In calcium carbonate‐treated plants, the lower starch accumulation, despite preserved thylakoid structure, suggests that the agglomerated calcium carbonate layer altered light penetration and internal light distribution, thereby reducing carbon assimilation efficiency. Residual oxidative stress—indicated by plastoglobule proliferation—may also have limited carbohydrate biosynthesis, further contributing to the reduced starch deposition in these leaves.

Anatomical adjustments under full sunlight may also influence foliar nutrient concentrations. Full‐sun control plants exhibited a thicker cuticle, consistent with increased structural investment and potentially higher leaf mass per area, which can shift nutrient concentrations on a dry‐mass basis through concentration/dilution effects (Riva et al. 2018). In this context, the higher K and Mg concentrations in full‐sun control plants may reflect stress‐driven changes in leaf structure and ion homeostasis. This interpretation is consistent with the central roles of K in osmotic adjustment and stomatal regulation and of Mg in photosynthetic metabolism, both of which can respond to light‐driven stress (Ahammed et al. 2022; Boaretto et al. 2020). Foliar concentrations of N, P, and Ca did not differ among treatments. Notably, neither kaolin nor calcium carbonate altered foliar Ca concentration, despite the latter containing Ca. This suggests that the films acted solely as surface barriers and did not provide immediate nutritional benefits or alter primary Ca uptake pathways.

The protective efficacy of particle films is supported by their ability to reflect a substantial portion of incident solar radiation, protecting leaves and fruits from high‐irradiance damage (Abreu et al. 2023). This reflectance can also enhance inner‐canopy photosynthesis by redistributing light after it strikes the coated surfaces (Liu, Henke, et al. 2022; Liu, Wang, and Chang 2022). We estimated that up to 45% of incident light was reflected by kaolin‐coated leaves (Figure 1). The high reflectance of these films mitigates the harmful effects of UV irradiance and elevated temperatures, improving CO_2_ assimilation and PSII performance (Bernardi et al. 2023).

The reflective capacity of particle films is influenced by factors such as suspension concentration, particle size, and dispersion (Abdel‐Aziz et al. 2022). In this study, kaolin particles were more finely and uniformly dispersed on the leaf surface under EDX imaging, whereas calcium carbonate formed thicker, more agglomerated deposits (Figure 2). Therefore, finer particles spread more efficiently and homogeneously, contributing to the higher reflectance observed in kaolin‐coated leaves.

Taken together, the anatomical and ultrastructural evidence reveals a coherent sequence of stress responses initiated by excessive energy input at the chloroplast level. As thylakoid integrity deteriorates and ROS proliferate, cellular energy and redox balance are challenged, which may be reflected in mitochondrial ultrastructural responses and altered ATP availability for repair processes. The resulting imbalance triggers lipid remodeling, plastoglobule expansion, and the activation of antioxidant pathways such as phenolic compound accumulation. These organelle‐level disruptions then manifest as broader tissue modifications—including cuticle thickening, altered palisade differentiation, and restricted CO_2_ diffusion—that progressively constrain photosynthetic performance. Under prolonged exposure, nutrient reallocation further reflects the metabolic cost of maintaining damaged cellular structures.

Particle films intervene at the very beginning of this cascade by reducing the irradiance load at the leaf surface. Kaolin, with its fine particle dispersion and high reflectance, interrupts the chain of injury more effectively than calcium carbonate, thereby preserving chloroplast organization and overall tissue structure, consistent with improved cellular homeostasis. This integrated conceptual framework clarifies not only how citrus leaves succumb to high irradiance but also how protective technologies can shift the thresholds of structural tolerance in an increasingly extreme climate. These damages explain the substantial crop losses observed in the Brazilian citrus belt in recent years.

## 5 Conclusions

High irradiance induces severe ultrastructural disruptions in citrus leaves, characterized by thylakoid disorganization, an increased plastoglobule formation, and alterations in oil cavities, all indicative of sustained photo‐oxidative stress. These injuries, first detailed and characterized here, are accompanied by anatomical modifications and shifts in foliar K and Mg concentrations, which may reflect stress‐driven changes in leaf structure and ion homeostasis under full sunlight. The preservation of cellular integrity in plants treated with kaolin or calcium carbonate particle films confirms excess irradiance as the primary driver of structural damage. Together, these findings establish an anatomical and ultrastructural reference framework that strengthens our understanding of citrus stress tolerance and supports the development of effective mitigation strategies in crop production under adverse high‐irradiance climate.

## Author Contributions

The study was designed and conducted by D.M., R.M.B., and L.G.P.B. L.G.P.B., J.P.M., and D.M. performed material preparation, data collection, and data analysis. L.G.P.B. and D.M. wrote the first draft of the manuscript. L.G.P.B., J.P.M., D.M., and R.M.B. commented on previous versions of the manuscript, and G.A.B. contributed to the final revision and preparation of the submitted version.

## Funding

This work was supported by the São Paulo Research Foundation (Grant 2020/05381‐6).

## Conflicts of Interest

The authors declare no conflicts of interest.

## Data Availability

The data supporting the findings of this study are available from the corresponding author upon reasonable request.
